# Complex Scenario of Homotypic and Heterotypic Zika Virus Immune Enhancement

**DOI:** 10.1128/mBio.01849-19

**Published:** 2019-09-03

**Authors:** Ernesto T. A. Marques, Jan Felix Drexler

**Affiliations:** a Department of Infectious Disease and Microbiology, University of Pittsburgh, Pittsburgh, Pennsylvania, USA; b Instituto Aggeu Magalhães, Fundação Oswaldo Cruz/MS, Recife, Pernambuco, Brazil; c Institute of Virology, Charité-Universitätsmedizin Berlin, corporate member of Freie Universität Berlin, Humboldt-Universität zu Berlin, and Berlin Institute of Health, Berlin, Germany; d German Centre for Infection Research (DZIF), associated partner Charité-Universitätsmedizin Berlin, Berlin, Germany; Vanderbilt University Medical Center

**Keywords:** Zika virus, antibody-dependent enhancement, congenital malformation, outbreak

## LETTER

We read the article by Shim and colleagues describing homotypic antibody-dependent-enhancement (ADE) of Zika virus (ZIKV) infections by low-level ZIKV-specific antibodies (1) with great interest. ADE predominantly describes a mechanism by which infectious antibody-virus immune complexes efficiently bind to Fc gamma receptors of immune cells, facilitating the infection of otherwise poorly susceptible cells. As noted by Shim et al., ADE has been described for genetically diverse viruses, including feline coronaviruses, human immunodeficiency virus type 1, and cytomegalovirus (2, 3). Although likely not solely responsible, ADE is a major component of the development of severe secondary dengue virus (DENV) infections (4). While a potential homotypic ZIKV-mediated ADE of subsequent ZIKV infections is of significant interest, several questions remain. The affinity, avidity, capacity of the antibody to bind to complement factor, and role of distinct epitopes in cell entry are all critical aspects determining whether viral immune complexes will be neutralized and destroyed or whether they will facilitate infection. In essence, even a potent neutralizing antibody can form enhancing immune complexes when it is present in low concentrations, as shown for DENV (4) and more recently for the genetically divergent arbovirus Chikungunya virus (5). Strikingly, we and others have recently shown that despite strong evidence for heterotypic ADE of ZIKV infection by DENV-specific antibodies *in vitro* and in murine infection models, prior DENV infection seems in fact to protect from both symptomatic Zika virus infection and congenital Zika syndrome (CZS) development *in vivo* (6–8). Whether the findings from Shim et al., whose data were raised *in vitro* and in experimental infections of immunodeficient mice, are clinically relevant thus remains unknown. Notably, our groups previously found that mothers of CZS cases had significantly higher, not lower, ZIKV-specific antibody titers than controls (9, 10). One the one hand, these data seem at odds with the experimental data reported by Shim et al. On the other hand, antibody titrations were performed months after ZIKV infection, and maternal antibody titers in CZS cases may have been boosted by prolonged Zika viremia potentially elicited by fetal-maternal ZIKV shedding (11). In any case, ZIKV spread rapidly throughout Latin America, infecting about 60% of the population in different regions (12, 13). A hypothetical homotypic ZIKV ADE is thus highly unlikely to have affected CZS development during the 2015–2016 Zika outbreak. The findings from Shim et al. may become relevant in the long- and medium-term perspectives on the fate of Zika in the Americas, when ZIKV-specific antibody titers drop to levels that may mediate enhancement. Immediate experimental assessments will have to consider the duration and strength of both humoral and cellular ZIKV- and DENV-specific immune responses and explore the immune interplay between the many flaviviruses endemic to Latin America.
